# Soft Tissue Vascular Anomalies of the Extremities: A Proposed Diagnostic Approach

**DOI:** 10.3390/life14060670

**Published:** 2024-05-23

**Authors:** Michele Fiore, Marta Bortoli, Andrea Sambri, Ludovica Lotrecchiano, Luigi Lovato, Michele Mirelli, Iria Neri, Massimiliano De Paolis, Bianca Maria Piraccini, Mauro Gargiulo

**Affiliations:** 1 Orthopedic and Traumatology Unit, IRCCS Azienda Ospedaliero-Universitaria di Bologna, 40138 Bologna, Italy; michele.fiore9@unibo.it (M.F.); marta.bortoli@ior.it (M.B.); massimiliano.depaolis@aosp.bo.it (M.D.P.); 2 Department of Medical and Surgical Sciences, Alma Mater Studiorum University of Bologna, 40138 Bologna, Italy; biancamaria.piraccini@unibo.it (B.M.P.); mauro.gargiulo2@unibo.it (M.G.); 3 Oncoematologic and Emergencies Radiology Unit, IRCCS Azienda Ospedaliero-Universitaria di Bologna, 40138 Bologna, Italy; ludovica.lotrecchiano@aosp.bo.it (L.L.); luigi.lovato@aosp.bo.it (L.L.); 4 IRCCS, Vascular Surgery Unit, IRCCS Azienda Ospedaliero-Universitaria di Bologna, 40138 Bologna, Italy; michele.mirelli@aosp.bo.it; 5 Dermatology Unit, IRCCS Azienda Ospedaliero-Universitaria di Bologna, 40138 Bologna, Italy; iria.neri@aosp.bo.it

**Keywords:** vascular malformations, diagnosis, vascular tumors, vascular anomalies

## Abstract

This narrative review aims to summarise the classification of vascular anomalies, their clinical presentation, and their radiological features to propose a diagnostic algorithm to approach patients with suspected soft tissue vascular anomalies of the extremities. The management of vascular anomalies necessitates a multidisciplinary approach. Clinical presentation and physical examination are sufficient in most cases to achieve a correct diagnosis. This is especially true for small congenital lesions of the skin and subcutaneous tissue. Imaging is used for accurate characterization of these lesions, especially in cases of atypical or vague clinical presentation, and to assess extension in cases of lesions that are larger and localized in deeper tissues.

## 1. Introduction

The definition of vascular anomalies (VAs) encompasses a spectrum of lesions that can be responsible for significant morbidity and mortality in both children and adults. Two main categories of lesions fall under the umbrella term of VAs: vascular tumors and vascular malformation; both are structural defects affecting the circulatory system, potentially involving both blood and lymphatic vessels. Even though they can occur in any tissue or anatomical location, they most commonly affect the extremities, in addition to the head and neck district [1,2,3].

Vascular malformations (VMs) are non-neoplastic structural anomalies, representing focal structural irregularities of the vascular system. VMs are typically present at birth and result from congenital errors in the development of blood vessels or lymphatic channels during embryogenesis. They are subcategorized considering the prevalent vasculature (arteries, veins, capillaries, lymphatics).

On the other hand, vascular tumors (VTs) are defined as a proliferation of neoplastic cells of endothelial origin [1,2]. They are further divided into benign, borderline and malignant forms [4]. These tumors can arise at any age and may either have slow and gradual or rapid and extensive growth [5]. Both VMs and VTs might be associated with several genetic syndromes, each bearing additional clinical features [6].

The diagnosis and management of these anomalies can be particularly challenging. Although a reliable and standardized classification system is essential to define the appropriate management strategy [7], some studies suggest that a significant percentage of patients affected by vascular lesions have been misdiagnosed [8]. This is probably because the origin and peculiarity of the different codified entities are not yet completely defined and inaccurate or unclear nomenclature has also been largely used in the literature, increasing confusion on the topic [9,10,11].

Patients affected by VAs can present for a first assessment to a variety of specialists; however, their management often necessitates a multidisciplinary approach, which involves collective efforts from dermatologists, interventional radiologists, pediatricians, plastic surgeons, orthopedic surgeons, vascular surgeons and geneticists.

It is paramount to facilitate cooperation among all the involved professionals, to have a shared vocabulary and to agree on the optimal workup to obtain a consistent and reproducible diagnosis. Reliable guidelines are essential to allow different specialists to coordinate their efforts in the evaluation and treatment of these patients. Nevertheless, there is still no consensus on the optimal diagnostic algorithm [12].

This narrative review aims to provide a brief overview of the classification of VMs, their clinical presentation and their radiological features to propose a pragmatic diagnostic algorithm to approach patients with suspected soft tissue vascular anomalies of the extremities and help navigate the diagnostic process.

## 2. Classification

Vascular anomalies were first subdivided into VMs and VTs by Mulliken and Glowacki in 1982 [4], who suggested distinguishing them into two groups based on the presence of proliferation and hyperplasia. Abnormal mitotic activity is the main histological feature of VTs, whereas VMs derive from angiogenesis dysregulation and do not show any active proliferation.

Although sporadic forms are the most frequent, hereditary forms linked to alterations to the angiogenic factors regulating the development of vessels during embryogenesis are also reported.

Built upon this fundamental principle, the International Society for the Study of Vascular Anomalies (ISSVA) classification system is the most recent and the most comprehensive [13] (Table 1). The last update, published in 2018, reported the known molecular and genetic markers to identify each entity for the first time.

The identification of genetic mutations specific to each entity—made possible by the widespread use of next-generation sequencing (NGS)—has provided new insights not only by providing markers to assist with the diagnosis but also by hinting at the pathogenetic mechanisms underlying these lesions. Two main molecular pathways are altered in vascular anomalies: the RAS/MEK/ERK pathway and the PIK3CA/Akt/mTOR pathway [14]. As will be further explored in the following paragraphs, PIKopathies mostly include venous and lymphatic malformations, whereas capillary malformations, arterio-venous malformations and vascular tumors fall under the category of RASopathies.

Some lesions still have not found their place in the classification and therefore remain listed as provisionally unclassified anomalies. The ISSVA classification will undoubtedly continue to evolve to include the new advances made especially in the molecular characterization of vascular anomalies.

### 2.1. Vascular Tumors

Vascular tumors (VTs) are defined as a proliferation of neoplastic endothelial cells. They can arise at any stage of life and may exhibit rapid growth. As illustrated in the 2020 WHO classification of soft tissue sarcomas [5], VTs are classified into benign, locally aggressive (or borderline), and malignant (Table 2). Most of them are benign [15].

#### 2.1.1. Benign Vascular Tumors

Benign VTs are quite frequent in the general population, often cutaneous, and mostly occur during childhood [16]. The most common histological types are infantile hemangiomas (IHs) and congenital hemangiomas (CHs).

IH affects 3–10% of newborns. They are inapparent at birth, start to become visible after a few weeks of life, and then fully develop during early infancy. Their clinical course is predictably characterized by a rapid proliferative phase, followed by a slow and gradual involution stage, which can last up to several years [17].

CH is significantly less common. As opposed to IH, CH is already visible in newborns. If a differential diagnosis becomes necessary, it can be made with molecular analysis; IH demonstrates hyperexpression of the GLUT1 transporter, which is not found in other similar lesions.

Other less common subtypes are listed in Table 2.

#### 2.1.2. Locally Aggressive Vascular Tumors

Locally aggressive VTs are rare entities. Among these, kaposiform hemangioendothelioma is the most frequent. Histologically, it stains positive for PROX-1, CD-34 and CD-31. It shares the GNA14 mutation with tufted angiomas, which is currently considered a different expression of the same entity. Kaposi sarcoma is caused by a Human Herpes Virus (HHV-8) infection.

#### 2.1.3. Malignant Vascular Tumors

Around 2% of vascular tumors demonstrate malignant biological behavior. Angiosarcomas and epithelioid hemangiomas are malign lesions, usually affecting older patients. They can originate from superficial tissues, but deeper lesions are highly suspicious for malignancy.

Angiosarcoma is composed of either hemangiomatous or lymphangiomatous cells. It can affect the superficial soft tissues (associated with chronic lymphedema) or deep tissues. It can be induced by radiotherapy or be associated with vascular or orthopedic implants [18]. It generally shows immunohistochemistry positivity to CD 34, CD 31, ki67.

### 2.2. Vascular Malformations

VMs derive from defects occurring during angiogenesis rather than from vascular proliferation; in fact, VMs demonstrate a normal cellular turnover (but an elevated proliferative potential).

VMs can be sporadic, but in a significant share of cases they exist as part of a syndrome. For the most part, VMs arise from post-zygotic mutations in an individual cell that then perpetuates itself and can be found in groups of cells throughout the body (creating mosaicism). In some cases, those mosaicisms can drive more complex somatic disorders that include, but are not limited to, vascular malformations. Furthermore, an even rarer occurrence is for VMs to derive from germline mutations, found in all tissues and organs; in these cases, they can either arise from a zygotic mutation or be inherited from a parent and are almost invariably associated with other anomalies [19].

VMs are classified into four types [20] (Table 3). Simple VMs are made of one type of vase and are further subclassified according to the prevalent component (capillary, venous, lymphatic, arteriovenous malformation, or fistulae). Multiple combinations of different vascular structures are described separately as the second type (combined malformations). Anomalies of major vessels (“channel type group”) usually affect certain large-caliber arterial, venous or lymphatic vessels, most of them stem vessels or central conducting vessels. They can affect the origin, course, number, length and diameter. They also include the presence of abnormal valves or intervascular communications, as well as the persistence of embryonic vessels. VMs associated with other anomalies are included in the fourth group.

#### 2.2.1. Capillary Malformations

Capillary malformations (CMs) are relatively common in the general population. They consist of dilated venule-type vessels, usually affecting the superficial dermis of the skin and mucous membranes. Abnormally formed capillaries are characterized by a chronically slower blood flow, which over time often results in soft tissue thickening and/or bone hypertrophy. CMs can present isolated or associated with other congenital anomalies in the context of complex poly-malformation syndromes.

The syndromes associated with CMs are reported in Table 4.

#### 2.2.2. Lymphatic Malformations

Lymphatic malformations (LMs) are the second most common type of vascular malformation, but they rarely affect the extremities [21,22]. LMs are lymphatic vessel anomalies, resulting in a range of different defects of the lymphatic drainage [23]. They can be classified as macrocystic, microcystic or mixed (macrocystic and microcystic). Lesions with dilations larger than 1–2 cm are usually defined as macrocystic, whereas finer alterations are described as microcystic.

Syndromes associated with LMs are reported in Table 5.

#### 2.2.3. Venous Malformations

Venous malformations (VeMs) are the most common vascular malformations of the extremities, with a prevalence in the general population of about 1%; they account for almost two-thirds of the total in lower extremities [24,25,26,27]. Histologically characterized by large vascular venous spaces, VeMs are mainly found in the skin and superficial soft tissues; however, VeMs tend to disregard anatomical boundaries and might therefore involve multiple tissues. As a side note, it is worth mentioning that they could develop primarily from intravascular bone vessels as well. The endothelium is immunoreactive for endothelial markers, e.g., CD31 and CD34.

The syndromes associated with VeMs are reported in Table 6.

#### 2.2.4. Arteriovenous Malformations

Arteriovenous malformations (AVMs) are anomalous direct communications between arterial and venous vessels; blood flow enters from feeding arteries and exits through draining veins, but these are connected via a nidus of multiple dysplastic vascular channels, among whose there are no capillaries [22,24,28]. This creates a hemodynamic effect that becomes larger the more significant the AVM.

On the one hand, without the balancing action of the capillary stream, AVMs create an area of minoris resistantiae, causing a drop in arterial pressure, subtracting flow from the bloodstream and potentially compromising the vascularization of the district downstream. On the other hand, the venous system receives an increased flow, and venous hypertension can become sufficiently significant to affect cardiac overload.

Syndromes associated with AVMs are reported in Table 7.

#### 2.2.5. Vascular Malformations Associated with Other Anomalies (Complex Vascular Malformations—CVMs) or Syndromic Vascular Malformations

Many vascular malformations can also present as an element of more complex sporadic or hereditary genetic alterations, which are listed in Table 4, Table 5, Table 6 and Table 7. Common features of those conditions include the possible coexistence of two or more different histological components, as well as possible multiple localizations. However, sometimes, VMs also coexist with non-vascular anomalies. This last occurrence is described as a separate category of the ISSVA 2018 classification, under the definition of complex vascular malformations. In particular, there is a rare genetic disorder group, the PIK3CA-related overgrowth spectrum (PROS), which is characterized by asymmetric overgrowth caused by somatic mosaic mutations of the PIK3CA gene. Other syndromes associated with growth anomalies but related to different mutations are described in Table 3.

### 2.3. Image-Based Classification

Despite the ISSVA classification, clinical and radiological features remain largely unlinked to the newly defined histological entities. The first radiologic classification of vascular anomalies has been proposed according to their Doppler signal on ultrasound investigation.

According to their flow dynamics, VMs can be subdivided into low-flow VMs (venous, lymphatic, capillary, capillary–venous, and capillary–lymphatic–venous) and high-flow VMs (arteriovenous malformations and arteriovenous fistulas).

Low-flow VMs (VeM, LM and CM are significantly more common, whereas high-flow malformations (mainly arteriovenous malformations and arteriovenous fistulas) account for about 10% of the total in the extremities [29].

Grouping VMs into high-flow and low-flow lesions is extremely useful in clinical practice since it can address the appropriate treatment even in the absence of a precise histopathological diagnosis [24].

## 3. Clinical Presentation

Soft tissue vascular anomalies can show extremely heterogeneous clinical presentations, with different features determined by the type, anatomical location and size of the lesion.

The most common cause for patients to seek medical attention is cosmetic concerns due to superficial lesions originating from the skin and other superficial tissues. However, vascular anomalies originating from deeper tissues might also determine severe functional impairment.

They commonly present with one of three typical clinical scenarios:

- A pigmented spot on the skin;
- A palpable soft tissue lump;
- Secondary clinical symptoms (pain, swelling and functional impairment).

### 3.1. Vascular Tumors

#### 3.1.1. Benign Vascular Tumors

Infantile hemangioma (IH) is the most common pediatric vascular tumor. The clinical course is typical: the lesion is not present in the first few days of life, but it becomes evident during the early neonatal weeks. The most common appearance is an elevated red papule or nodule with a sharp margin, demonstrating a rapid proliferative phase during the first 3–6 months after birth [30]. After a period of relative stability, over the following years, IH eventually regresses. Superficial IHs exhibiting classical characteristics are easily identified and diagnosed by clinical examination alone; however, they sometimes present as deep masses, difficult to distinguish from other more concerning lesions, and therefore require a US examination. Once the diagnosis has been established, they generally do not require any treatment [31]. In children with IH at risk of complications, oral propranolol is the first-line treatment. Complications that determine an indication for treatment include a threat to survival (high-output heart failure or obstructive/compressive compromise of the respiratory tract), functional or aesthetic damage to surrounding structures and ulcerated lesions.

Multifocal IHs are more likely to be associated with infantile hepatic hemangioma; large facial IHs warrant investigation for PHACE complex syndrome (posterior fossa malformation, hemangiomas, arterial, cardiac, and eye anomalies). If a cutaneous IH is distributed in the “beard” area, an associated airway IH that usually involves the subglottis must be excluded.

Congenital hemangiomas (CHs), conversely, are significantly less common [32]. Their presentation can be similar to IH, despite producing more bluish-red lesions, with less-well-defined margins and a pale rim. The differential diagnosis is guided by the history since CHs are fully developed at birth and do not increase in size over the first weeks of life; based on their clinical evolution, CHs can be distinguished as rapidly involuting (RICH), partially involuting (PICH) and non-involuting (NICH) [33].

#### 3.1.2. Locally Aggressive Vascular Tumors

Tufted angioma and kaposiform hemangioendothelioma are locally aggressive tumors with very low metastatic potential. They typically affect children and the extremities represent their most typical location [34]. The clinical appearance is rather characteristic: a red-blue mass without sharp margins and hard and not compressible to the touch, with almost constant skin involvement and concomitant purpura and ecchymoses. When they are larger, they can cause compression symptoms and determine lymphedema. Surrounding tissue infiltration is not uncommon, with potential erosion of the adjacent bone. Large lesions (>5 cm) can be accompanied by systemic symptoms, of which the most serious is the Kasabach–Merritt phenomenon (KMP), which represents a consumption coagulopathy caused by the adhesion, sequestration and activation of platelets inside the tumor, often associated with secondary hemolytic anemia. It can be extremely severe and lead to exitus if not promptly recognized. Clinically, it should be suspected if the tumor shows a rapid increase in size, suddenly becomes painful or if a purpura appears (all signs of intralesional thrombosis).

Kaposi sarcoma can affect all organs and tissues, leading to a wide variety of clinical manifestations ranging from patches or nodules on the skin or mass-like lesions in deeper tissues. It can determine osteolytic lesions of nearby bones with a metastasis-like appearance.

#### 3.1.3. Malignant Vascular Tumors

Malignant vascular tumors are only occasionally reported in children, and they typically affect adults. Their clinical presentation depends on the site of occurrence, generally presenting as deep palpable lumps. This clinical finding is highly suggestive of a malignant lesion and therefore requires imaging work-up.

### 3.2. Vascular Malformations

Contrary to vascular tumors, VMs are present at birth and tend to increase in size harmonically with the patient’s growth. They are usually non-proliferative and do not undergo spontaneous regression. Even though they are present at birth, VMs can be missed in the early stages of life and become clinically symptomatic depending on their location and size during adolescence or adulthood. Moreover, VMs often demonstrate sudden enlargement after trauma or in conjunction with hormonal changes, such as puberty and pregnancy [15,35,36].

#### 3.2.1. Capillary Malformations

CMs are the most common low-flow VMs. In consideration of their peculiar cutaneous manifestations, they rarely require imaging to confirm the diagnosis [15]. They present as bright pink, red or purple patches and were previously known as “port-wine stains”. Color changes can occur during growth, as they often darken with age.

Sometimes, they develop blebs and might bleed. It is not uncommon for CMs to develop on top of deeper vascular malformations and to be therefore associated with some degree of deep tissue involvement. In these cases, physical examination can underestimate their full extent.

Furthermore, CMs affecting the limbs can sometimes present in association with hypertrophy and/or hypermetry of the affected limb.

#### 3.2.2. Lymphatic Malformations

LMs are extremely rare at the extremities, as they usually develop in the cervico-facial region or in proximity to large lymphatic trunks. They present as soft compressible masses or swellings, with usually undamaged, skin-colored overlying skin. They are rarely painful unless other structures become compressed during their growth. The most common complication causing them to become acutely symptomatic includes endo-cystic hemorrhage, manifesting with an acute volumetric increase, fever, local pain, and compression signs.

#### 3.2.3. Venous Malformations

VeMs can present as either small, well-circumscribed lesions or large, infiltrative ones [13,23]. They can be observed in any anatomical location, with a preference for the limbs, and can either be superficial or deep, intramuscular and even intraosseous. Superficial VeMs are blue-purple lesions and collapsible, with a soft consistency; deep forms’ presentation is more subtle, with generalized swelling and pain. Hemodynamic changes and chronic venous insufficiency become more frequent the larger the lesion, leading to edema, stasis dermatitis and even skin ulceration.

It is not uncommon to observe an association of anomalies of bone vascularization and hypertrophy or hypotrophy of the affected skeletal segment (vascular bone syndrome). In some cases, they can arise from inside a joint. The presence of an intraarticular mass can determine symptoms like mechanical pain, swelling and joint stiffness. Furthermore, lesions prone to bleeding can determine recurrent hemarthrosis and degenerative pathology of the joint, eventually determining deformities similar to those seen in hemophiliac patients [37].

VeMs are associated with localized intravascular coagulopathy (LIC) in about 40% of cases, occurring when the stagnant blood flow inside and surrounding the lesion results in activation and consumption of platelets and coagulation factors.

#### 3.2.4. Arteriovenous Malformations

AVMs, because of their arterial component, constitute the paradigm of high-flow malformations. Because of their pathophysiology and their systemic impact, they can look very different in different clinical stages. In an initial stage, AVMs can be asymptomatic or be recognized as warm palpable masses, sometimes with a thrill or bruit [36]. As they increase in size, pain becomes more common, along with compression neuropathy. Eventually, when the compensating mechanisms fail, signs of venous insufficiency become apparent (edema, skin discoloration and ulceration, eventually leading to hemorrhages due to the rupture of superficial veins). In late phases, venous overload can eventually lead to cardiac failure in extreme cases [31]. These stages are reported in Table 8 [38].

## 4. Imaging

Imaging plays a crucial role in identifying and classifying VA, defining their extent, and helping to decide the treatment protocol [39]. Imaging is used for verification, particularly if the clinical presentation is atypical or unclear [15]. It is usually required for an accurate characterization of VAs, especially in cases of lesions that are larger and localized in deeper tissues [40,41].

### 4.1. Ultrasound

Ultrasound imaging (US) represents the first-line examination; it is particularly useful for superficial lesions and in pediatric cases.

US offers several advantages, which include the lack of ionizing radiation, unnecessary sedation in small children, cost-effectiveness, and wide disposal. Moreover, grayscale US together with color and spectral Doppler can be used for accurate diagnosis in most cases when read in combination with clinical features. Color and power Doppler US can help to define the hemodynamics of VAs [19]. Disadvantages include operator dependence and incapacity to assess deep lesions satisfactorily [42,43].

**Figure 1:**
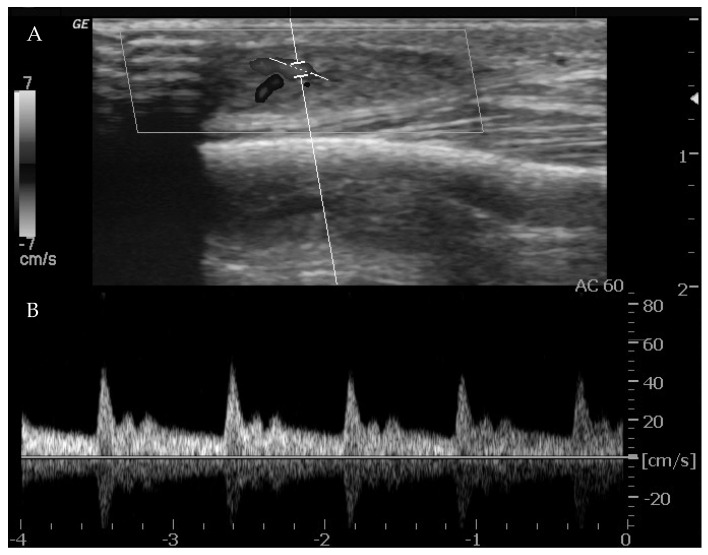
Ultrasound imaging with power Doppler on the thigh. (**A**). A well-defined, solid, ovoid lesion is seen in the subcutaneous plane, without calcification or cystic changes. (**B**). Significant flow is noted on color Doppler with an artery entering and exiting the lesion.

Ultrasounds can provide indications of the exact type of lesion and can help to distinguish it from other lesions. It is also possible to gather information on the morphology (size, anatomical location and depth), number and compressibility (hard, non-compressible lesions are always suspicious for a malignant nature) [44]. It can also discriminate high-flow from low-flow anomalies and frequently offers a specific diagnosis when there are characteristic features of VMs (4, 10, 15, 18, 20). The presence of arterial flow is suggestive of high-flow lesions such as hemangiomas, other vascular tumors and high-flow VMs (Figure 1).

When arterial flow is recognized on Doppler US, further considerations can be made. High vessel denseness and high Peak Systolic Velocity (PSV) can be recognized. More information can be gained by calculation of resistive index: the arteries inside high-flow VA show low-resistance flow, with low values of resistive index (RI).

However, it must be considered that one or a few areas of arterial flow could also be observed within low-flow VM, e.g., venous and lymphatic malformations which frequently infiltrate around normal structures, and a few arterial vessels might be seen passing through them. Also, lymphatic malformations can show areas of arterial flow inside the septa/walls of their cystic component. These foci of arterial flow inside low-flow lesions show high RI values, in contrast to arteries within high-flow lesions [43].

The imaging findings of high-flow vascular tumors (e.g., congenital hemangiomas) are similar to those of high-flow VM; however, high-flow VTs have a lobular morphology and an enhancing soft tissue component.

Intralesional soft tissue is appreciated in VTs, whereas lesions without any soft tissue part are typical of high-flow malformations. The presence of a soft tissue area is the most reliable predictor for differentiation of hemangiomas (or other VTs) from arteriovenous malformations [45].

If venous flow is observed, the lesion can be a VeM. On Doppler US, VeMs are frequently hypoechoic; they can infiltrate multiple tissue planes or can be well-marginated. Phleboliths, if present, are typical [43]. However, not all VeMs show any flow on Doppler US. In case of no flow detection, the lesion may be either a lymphatic or a venous malformation (with very slow flow or thrombosis). Also, it has to be considered that approximately 16% of all VeMs show no detectable flow on Doppler US [46]. 

Contrast-enhanced US (CEUS) can be useful for visualizing poorly defined lesions; it can also have some advantages in image-guided procedures [21]. The addition of a microbubble contrast medium can enhance the visualization of small arteriovenous shunts and low-flow vessels compared to US with Doppler [22].

### 4.2. X-rays

**Figure 2:**
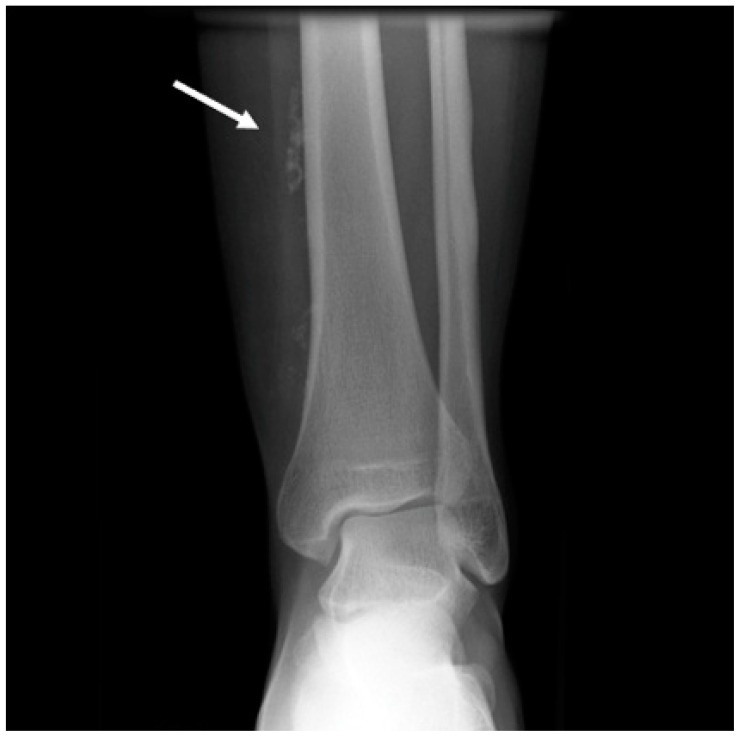
Anteroposterior X-rays showing phlebolites (white arrow) within soft tissues near the tibial diaphysis.

A standard radiograph is not part of the diagnostic workup of VAs, but it can be useful as the initial imaging step for patients who present with aspecific extremity complaints. It helps to rule out other more common causes of pain and deformity in the extremities. Radiographs may be normal or can show a soft tissue mass [35,42]. Phleboliths (areas of spontaneous thrombosis) might also be observed, thus providing a clue for the diagnosis of VeMs and hemangiomas (Figure 2). They are reported in a variable range between 20% and 67% of VeMs and hemangiomas [15].

### 4.3. Magnetic Resonance Imaging

Magnetic Resonance Imaging (MRI) is the gold standard technique thanks to its excellent soft tissue contrast resolution [47]. It can evaluate the entire extent of the lesions and their relationship with the contiguous structures and can help to classify the lesions based on their enhancement characteristics on gadolinium-enhanced MRI.

Time-resolved MRI angiography has become an important tool for the comprehensive evaluation of VAs. They can provide information about the hemodynamics of VAs, thus enabling differentiation between high-flow and low-flow VMs.

The standard sequences for characterization and diagnosis of suspected VMs include spin echo (SE) or fast SE (FSE) T1-weighted imaging (T1WI) for regional anatomy, and SE T2-weighted imaging (T2WI) with and without fat saturation to delineate the extent of the lesion. Gradient recalled echo (GRE) T2* WI is able to differentiate areas of high-flow or hemosiderin deposition from bleeding [48].

Contrast-enhanced MR angiography (MRA), performed with a 3D T1-weighted fast gradient echo (GRE) sequence is necessary to evaluate the perfusion of the lesion. Usually, imaging is performed in the arterial phase and several venous phases; images are also obtained before contrast material administration for posterior subtraction of contrast-enhanced images and 3D reformation. For complete assessment and classification of the lesion, dynamic time-resolved MR angiography, with its high temporal resolution, enables clear separation of arterial inflow from venous drainage and the detection of early venous shunting. Also, it allows for the acquisition of information about the contrast material arrival time (the interval between the onset of enhancement and the maximal percentage of enhancement in the vessels) and flow direction; furthermore, it reduces motion artifacts [28,49]. The angiographic study should be followed by post-contrastographic FSE T1-weighted imaging to assess post-contrast anatomy.

**Figure 3:**
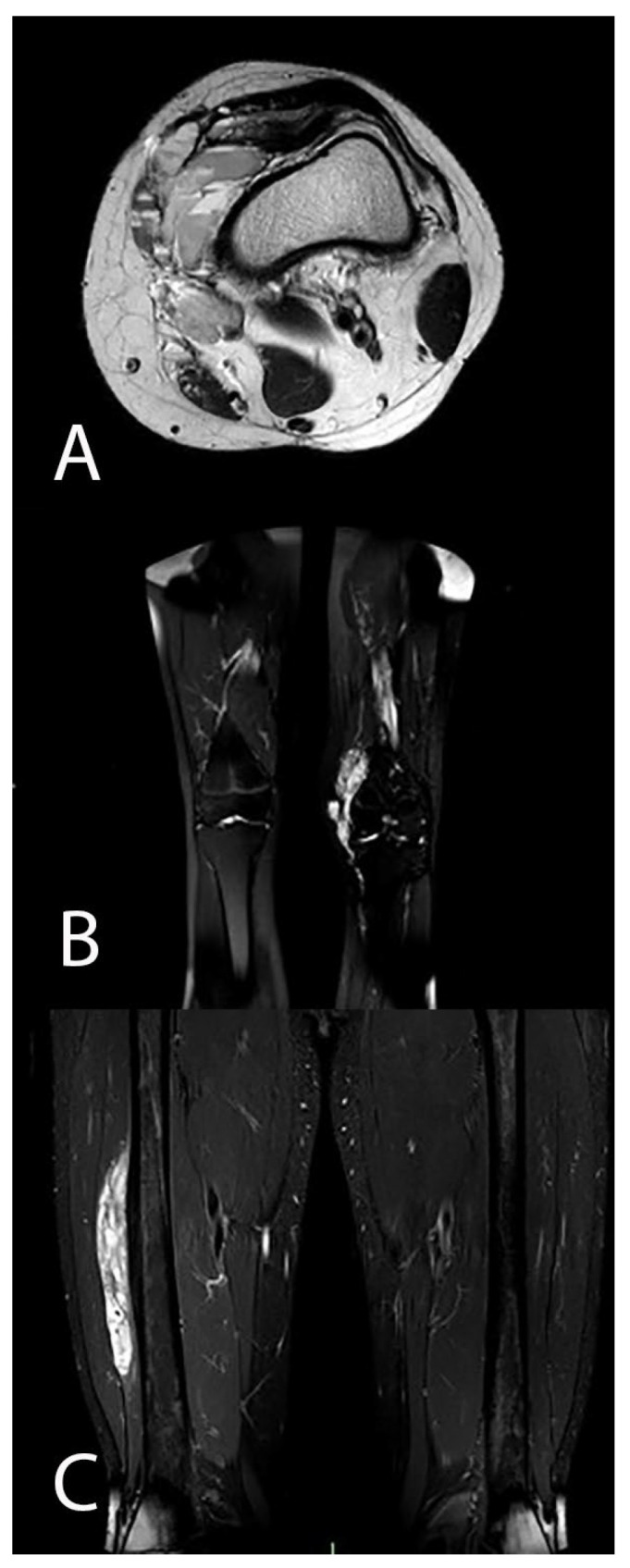
(**A**). Axial view T2 sequence MRI showing increased signal of a soft tissue MAV near the medial region of the knee, showing some fluid–fluid level related to possible hemorrhage or high protein content. (**B**). Coronal view T2 fat sat sequence MRI demonstrating hyperintensity of the signal of a soft tissue venous malformation in the medial region of the knee, with some intralesional low-intensity septa. T2 sequences help define the extent of the lesion. (**C**). Coronal view STIR sequence MRI shows high hyperintensity of the signal of an intramuscular venous malformation adjacent to the femoral diaphysis.

Venous malformations are usually septated lesions, with intermediate to decreased signal intensity on T1-weighted images and increased signal intensity on T2-weighted and STIR images (Figure 3).

Hemorrhage or high protein content may cause internal fluid–fluid levels. Heterogeneous signal intensity can be observed on T1-weighted images in cases of thrombosis or hemorrhage. The detection of phleboliths, which appear as small foci of low signal intensity in all pulse sequences, can also help with diagnosis. Fat-suppressed T2-weighted and STIR images can exquisitely define the extent of VeMs [28,50].

The diagnosis of a low-flow VM is based on the absence of flow cavities on FSE images. Sporadically, low signal intensity patterns, septa, thrombosed vessels, or phleboliths can mimic flow cavities on cross-sectional images. Contrast-enhanced and GRE images can help to distinguish these other causes of low signal intensity from flow-related signal cavities. Phleboliths and calcifications typically appear as signal voids with all pulse sequences; on the other hand, signal voids related to high flow exhibit enhancement and appear as high signal intensity foci on GRE images [49]. After injection of gadolinium, VeMs are characterized by lack of arterial and early venous enhancement and absence of enlarged feeding vessels or arteriovenous shunting. They characteristically show slow gradual filling with contrast material and can display typical nodular enhancement of tortuous vessels on delayed venous phase images [51]. Delayed post-contrast T1-weighted images of VeMs frequently show diffuse enhancement of the slow-flowing venous channels [52].

The appearance of lymphatic malformations on FSE T1w and T2w or STIR sequences can be similar to VeMs; nevertheless, after contrast injection, there is no significant enhancement of microcystic lymphatic malformations [53], whereas macrocystic lymphatic malformations exhibit only rim and septal enhancement and no central filling of the cystic structures [24].

MR imaging findings comprise high-flow tortuous and enlarged feeding arteries and draining veins which appear as large flow voids on SE images or high signal intensity foci on GRE images, in the absence of a well-defined mass. Intraosseous extension of the lesion can be seen as decreased marrow signal intensity on T1-weighted images [49,54]. Gadolinium enhancement is fundamental in evaluating the feeding arteries and draining veins. The dynamic opacification of the arteriovenous malformations is well assessed by using time-resolved dynamic 3D MR angiography; normally, early venous filling is typically seen in AVMs.

### 4.4. Computerized Tomography

Contrast-enhanced multi-slice computerized tomography (CT) allows for rapid evaluation of VMs with detailed assessment of the feeding and draining vessels. The main drawback of CT is the important dose of ionizing radiation, which is mainly significant in children. In detail, time-resolved CT arteriography and CT venography (4D CT imaging) are generally suggested when MRI is contraindicated.

CT may reveal a soft tissue mass with or without phleboliths, as well as provide information about size and lesion size [31]. Bone involvement and acute complications like hemorrhage can also be evaluated with CT [4,16]. Intravenous contrast administration improves the definition of the lesion and the assessment of enhancement patterns, which may help in the differential diagnosis [4]. It is usually more useful for high-flow lesions, such as an arteriovenous malformation. It can outline the feeding arteries, nidus, and draining veins, which typically characterize these lesions [32]. It may also provide additional information on the lesion extent and invasion into muscular compartments and bones [32]. CT may also be useful in excluding some other soft tissue masses such as lipomas, which are well-demarcated and display low-density attenuation [55].

### 4.5. Molecular Imaging

Molecular imaging techniques such as fluorodeoxyglucose PET/CT can be used for the diagnosis of some VTs [17]. The development of new radiotracers can assist with the diagnosis of certain VMs and in the quantification and assessment of response to systemic therapy [56].

### 4.6. Arteriography

There is no evidence to support the use of arteriography as the initial imaging evaluation for a suspected VM because of its invasiveness. Moreover, MRA is noninvasive and can depict the vascular anatomy of a malformation nearly as well as arteriography [57], making it the favorite second-line imaging method. Arteriography provides the highest resolution imaging of small vessels and superior temporal resolution for the assessment of flow dynamics. Thus, it can be advantageous for high-flow lesions when MRA findings are ambiguous or when the highest-available vascular detail resolution and/or better estimation of intralesional shunting is needed in planning treatment [35,36].

### 4.7. Differential Diagnosis: Benign vs. Malignant

Malignant vascular tumors are rare, accounting for <1% of all sarcomas; although the definitive diagnosis will always be based on histology, imaging can help distinguish benign and primary vascular malignant tumors.

As far as vascular tumors are concerned, benign lesions tend to be included in the low-flow group, whereas malignant lesions are usually more solid and lack flow altogether. In some instances, tumor vessels (for example in angiosarcomas) might be so well represented that they exhibit high flow.

Most malignant vascular tumors have a predominantly extravascular growth pattern, even if an intravascular variant exists; imaging such as contrast-enhanced CT or MRI can be useful in differentiating the two variants [58].

The MRI feature of hemangioblastomas is a cystic structure containing a mural nodule with marked contrast enhancement. Contiguous flow cavities can be observed, which represent the feeding and draining vessels. Large hemangioblastomas are heterogeneous on T2-weighted and gadolinium-enhanced sequences; small lesions are generally more homogeneous [59].

Differentiation among hemangioendothelioma, hemangiopericytoma, and angiosarcoma is challenging. On MRI, a typical finding of hemangioendothelioma is a brightly enhanced soft tissue mass, often hyperintense on T2WI, with prominent flow voids.

Angiosarcoma can show more aggressive destruction with osseous invasion and a permeative pattern with a soft tissue component. CT can identify bone involvement; CT and MR imaging may reveal nonspecific marrow involvement with or without soft tissue extension. On MRI, lesions can be similar to hemangiopericytomas, showing brightly enhanced soft tissue mass, frequently hyperintense on T2WI, with prominent flow cavities [60]. Prominent serpentine vessels can be recognized in some cases, thus suggesting these diagnoses. These vascular channels are more often in the periphery of the mass and are particularly prominent in hemangiopericytoma, reflecting the vascular supply.

## 5. Proposed Diagnostic Approach

There have been several attempts at creating a systematic approach to vascular anomalies, but no one has received large-scale validation. Most of those proposed frameworks are still incomplete or focus on aspects specific to a single medical specialty.

Clinical presentation and physical examination are sufficient in most cases to achieve a correct diagnosis. This is especially true for small congenital lesions of the skin and subcutaneous tissue.

**Figure 4:**
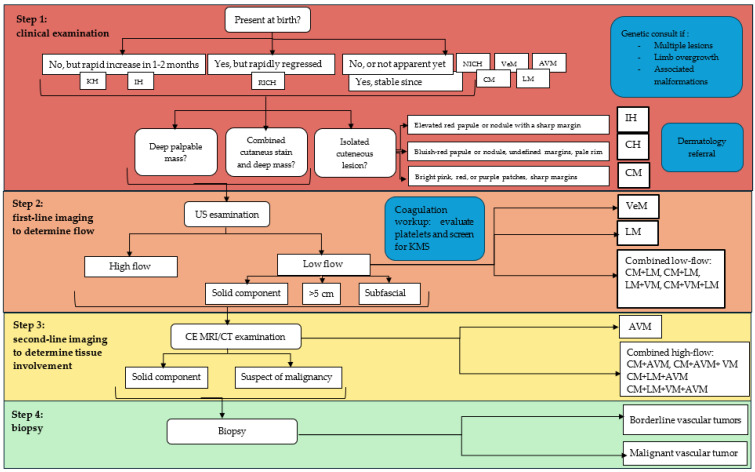
Summary table illustrating the proposed algorithm.

Imaging is used for accurate characterization of these lesions, particularly if the clinical presentation is atypical or vague [15,61], and to assess extension in cases of VA which are larger and localized in deeper tissues [40,41] (Figure 4).

### 5.1. Step 1: History and Clinical Examination

Soft tissue vascular anomalies can manifest either as cutaneous lesions, deep lumps or secondary clinical expressions due to localized or systemic influence of the primary lesion.

A preliminary clinical approach should aim, on the one hand, to identify lesions which do not need further exams and, on the other hand, to alert the clinician to associated conditions that should be further investigated. We suggest that, in addition to accurate description of the size, location, number and appearance of the lesions, there might be two main items to consider during the initial evaluation.

The first aspect to assess is the time of the presentation. It can be useful to recognize four different patterns, described as follows:

(1) Present at birth, stable since then or growing harmonically with the child.

Typical presentation for most vascular malformations, including CMs, VeMs, AVMs, LMs; some forms of congenital hemangiomas, defined as non-involuting (NICH), can also present similarly.

(2) Present at birth, but rapidly involuting.

Suggests rapidly involuting congenital hemangioma (RICH).

(3) Not present/apparent at birth but demonstrating rapid growth during the first 1–2 months of life.

Highly suspicious of infantile hemangioma (IH) or kaposiform hemangioendothelioma (KH).

(4) Not present/apparent at birth, becoming recognizable later in life (childhood, adolescence, adulthood).

Could be a mimic for any lesion in pattern 1.

Second, it might be useful to look for typical clinical characteristics supporting one of the shortlisted differential diagnosis. In particular, there are three VAs that are usually diagnosed on physical examination alone, whose morphological features are peculiar:

(1) CM: bright pink, red or purple stains (previously known as port-wine stains).
(2) Superficial IH: elevated red papule or nodule with a sharp margin.
(3) Superficial CH: bluish-red papule or nodule, undefined margins, pale rim.

IH and CH demonstrating a deep component, conversely, should always undergo further investigations, as should all VAs presenting as deep soft tissue masses or those with an atypical appearance.

The presence of multiple lesions and associated specific features may suggest the possibility of syndromic disease and the need for genetic consultation.

### 5.2. Step 2: First-Line Imaging (US)

Ultrasound examination can identify the flow dynamics inside the lesion, thus highlighting any sign of arterial-like flow. US examination is indicated in all VAs that cannot be diagnosed via clinical history and examination alone. Nonetheless, it is necessary to be aware that US can fail to visualize or can underestimate the extent of lesions that are deep, particularly large ones or those in fastidious anatomic sites. It might be sensible to apply the same criteria that have been validated for soft tissue tumors, extending indication to further imaging to lesions that are subfascial and/or larger than 5 cm in diameter.

At this stage, lesions will be grouped into low-flow and high-flow. Low-flow lesions located in regions appropriately explorable via US can be accurately identified. VeMs and LM can be distinguished via morphological features and the presence/absence of venous flow. Every time a low-flow lesion is diagnosed, the patient should undergo a coagulation workup to rule out LIC, Kasabach–Merrit syndrome, and other coagulopathies. Indications for second-line imaging exams include:

- Arterial flow (high-flow lesions) or unclear flow dynamics;
- Significant solid components;
- Lesions larger than 5 cm, subfascial lesions.

### 5.3. Step 3: Second-Line Imaging (CE-MRI or CT-Angio)

Contrast-enhanced MRI or angio-CT are indicated to evaluate the local extent of larger, deeper lesions. In case of high-flow lesions, they can also confirm the diagnosis and to guide the choice of treatment.

The use of routine MRI in low-flow lesions has been proven to increase costs without improving patient care [62].

### 5.4. Step 4: Biopsy

Histopathologic examination of a vascular anomaly is a delicate procedure (in consideration of the high risk of bleeding) and it is rarely necessary. However, lesions suspected of malignancy should always undergo a biopsy.

## 6. Conclusions

Under the definition of vascular anomalies lies a wide variety of different conditions, ranging from self-limiting and/or asymptomatic birthmarks to life-threatening conditions. Some vascular lesions, such as hemangiomas and capillary malformations, are common in the general population, whereas others are extremely rare and are very challenging to correctly diagnose and treat.

Although great progress has been made over the past decade, a systematic approach to diagnose and treat these lesions has still not been standardized. In this narrative review, we proposed a simple but comprehensive algorithm that could be applied to navigate this intricate field. Vascular anomalies should be managed in a multidisciplinary setting and centralized to reference centers to be managed by dedicated teams.

## Figures and Tables

**Table 1:** Overview of vascular anomalies classification, ISSVA 2018.

Vascular Anomalies				
Vascular Tumors	Vascular Malformations			
Benign	Simple	Combined	Of major named vessels	Associated with other anomalies
	Capillary malformation	Combination of two or more simple malformations in the same lesion	Anomalies of origin, course, number, length of major, persistence of embryonal vessels	Syndromes in which vascular malformations are associated with other symptoms
Borderline (locally aggressive)	Lymphatic malformation			
	Venous malformation			
Malignant	Arteriovenous malformation			
	Arteriovenous fistula			

**Table 2:** Vascular tumors, with identified genes involved. ISSVA 2018.

Vascular Tumors					
Benign		Borderline		Malignant	
Infantile hemangioma (IH)	Possibly VEGFR2 and TEM8	Kaposiform hemangioendothelioma	GNA14	Angiosarcoma	VEGFR2 VEGFR3 MYC amplification
Congenital hemangioma (CH)	GNAQ/GNA11	Retiform hemangioendothelioma		Epithelioid hemangioendothelioma	CAMTA1/ TFE3
Rapidly involuting (RICH)		Composite hemangioendothelioma		Other	
Non-involuting (NICH)		Pseudomyogenic hemangioendothelioma	FOSB		
Partially involuting (PICH)		Polymorphous hemangioendothelioma			
Tufted Angioma	GNA14	Hemangioendothelioma NOS			
Spindle-cell hemangioma	IDH1/IDH2	Kaposi sarcoma			
Epithelioid hemangioma	FOS	Others			
Pyogenic granuloma	BRAF/RAS/GNA14				

**Table 3:** Vascular malformations. ISSVA 2018.

Vascular Malformations					
Simple		Combined	Of Major Named Vessels	Associated with Other Anomalies	
Capillary malformation	GNAQ/GNA11, RASA1, EPHB4	Capillaro-venous (CVM)	Origin anomalies	Klippel–Trenaunay syndrome	PIK3CA- related overgrowth spectrum (PROS)
Lymphatic malformation	PIK3CA	Capillaro-lymphatic (CLM)	Course anomalies	MCAP syndrome	
Venous malformation	TIE2/TEK/PIK3CA	Lympho-venous (LVM)	Number anomalies	CLOVES syndrome	
Arteriovenous malformation	MAP2K1/MEK1/KRAS NRAS/BRAF	Capillary-lympho-venous (CLVM)	Length anomalies	CLAPO syndrome	
		Capillary-artero-venous (CAVM)	Diameter anomalies	Parkes Weber syndrome	Other syndromes associated with growth anomalies
		Capillary-lympho-artero-venous (CLAVM)	Valves	Servelle–Martorell syndrome	
			Abnormal communication (fistula)	Sturge–Weber syndrome	
			Persistence of embryonal vessel	Diffuse capillary malformation with overgrowth (DCMO)	
				Maffucci syndrome	
				Proteus Syndrome	
				Bannayan–Ryley–Ruvalcaba Syndrome (PTEN hamartoma syndromes)	

**Table 4:** Syndromes associated with CMs. ISSVA 2018.

Syndromes Associated with CMs	Symptoms	Genetic Mutation
Cutis marmorata telangiectasia (CMTC)	Cutis marmorata: congenital reticulated bluish mottling of the skin: - Darker in the cold or while crying; - Disappears during the first year of life.	Possibly GNA11
	Segmental localization	
	Hypertrophy of affected limb	
Sturge – Weber syndrome (SWS)	Facial CM associated with ocular anomalies (glaucoma, choroidal vascular anomalies)	GNAQ
	Vascular anomalies of the meninges	
	Seizures, developmental delay, contralateral hemiplegia	
	Soft tissue overgrowth (55–70%), and skeletal hypertrophy (maxilla)	
Phacomatosis pigmentovascularis	Red/brown nevi present at birth	GNA11/GNAQ
	Glaucoma	
	Seizures	
	Cognitive delay	
	Pigmented spots on the sclera	
Capillary malformation–arteriovenous malformation (CM–AVM)	Circumscribed, small diffusely distributed CMs (6% single lesion); halo surrounding	RASA1/EPHB4
	AVM lesions (seen in 80%, intra- or extracranial)	
	May be associated with vein-of-Galen aneurysm	
Generalized essential telangiectasia	Generalized telangiectasias that progress peripheral to central	None identified
	Associated with mild pruritis, numbness, tingling or burning	
	Conjunctival/mucosal telangiectasia	

**Table 5:** Syndromes associated with LMs. ISSVA 2018.

Syndromes Associated with LMs	Symptoms	Genetic Mutation
Gorham–Stout syndrome, aka “disappearing bone disease”	Progressive osteolysis with replacement of bone by soft tissue and vascular channels, primarily lymphatic in origin	Unknown
	Defining characteristic is the disappearance of bone rather than a mass lesion in bone	

**Table 6:** Syndromes associated with VeMs. ISSVA 2018.

Syndromes Associated with VeMs	Symptoms	Genetic Mutation
Fibroadipose vascular anomaly (FAVA)	Intramuscular; increased fibroadipose tissue and smaller, non-spongiform vessels	PIK3CA
	Often single limb, appears slightly enlarged	TEK/TIE2
Blue rubber bleb nevus syndrome	Cutaneous and mucous VeM (skin, GI tract)	
	Visceral VeM (liver, spleen, eye...)	
	Multiple bluish or violaceous lesions, compressible and hyperkeratotic	
	Patients are often born with one dominant lesion and develop more during their lifetime	
PTEN-associated venous anomalies	Hamartoma of soft tissue, intramuscular vascular lesions, with fast-flow lesions in 86%	PTEN
	Increased risk of other tumors	

**Table 7:** Syndromes associated with AVMs. ISSVA 2018.

Syndromes Associated with AVMs	Symptoms	Genetic Mutation
Hemorrhagic Telangiectasia (HHT) Osler–Weber–Rendu Syndrome	Telangiectasias (lip, tongue, buccal mucosa, face, chest, and fingers)	ENG, ALK1/ACVRL1, GDF2 SMAD4/MADH4
	AVMs throughout multiple sites including cerebral, pulmonary, gastrointestinal, and hepatic	
	Hemorrhagic symptoms (epistaxis, GI bleeds)	
CM-AVM	Small, multifocal CMs	RASA1 EPHB4
	30% of cases associated with AVMs	

**Table 8:** Schobinger staging of AVMs.

Stage		Clinical Feature	Vascular Alteration
I	Quiescence	Warm, pink-blue	Arterio-venous shunt
II	Expansion	Enlargement, pulsation, thrill, bruit	Tortuous downstream veins
III	Destruction	Dystrophic skin changes, ulceration, bleeding, pain, infection	Ischemia of surrounding tissue
IV	Decompensation	Cardiac failure	Systemic volume overload

## Data Availability

Data used in this article are available in literature.
